# A Systematic Review of Mobile Apps as an Adjunct to Psychological Interventions for Emotion Dysregulation

**DOI:** 10.3390/ijerph20021431

**Published:** 2023-01-12

**Authors:** Federico Diano, Luigia Simona Sica, Michela Ponticorvo

**Affiliations:** Department of Human Studies, University of Naples “Federico II”, 80138 Naples, Italy

**Keywords:** mobile application, psychotherapy, mental health, emotion dysregulation

## Abstract

Background: Mental health care has been enriched with the progressive use of technology during the last ten years, in particular after the COVID-19 pandemic. Mobile applications (apps) and smartphones have become the most widespread access point for many people who look for self-help in the psychological domain. Objective: We focused on a systematic review of mobile apps for mental health, focusing on the blending of apps with psychotherapy contexts, with a specific focus on emotional dysregulation. Methods: A comprehensive literature search (January 2017 to August 2022) in PubMed, PsycInfo, Web of Science, and the Cochrane Library was conducted. Abstracts were included if they described mental health mobile apps targeting emotional dysregulation and their use during ongoing psychological or psychotherapy treatment for adults and adolescents. Results: In total, 397 abstracts were identified; of these, 19 publications describing apps targeting borderline personality disorder, depression, anxiety, suicidal behaviors, and post-traumatic stress disorders met the inclusion criteria. Conclusions: App-enhanced psychotherapy might be a winning combination in many scenarios, but at the same time, many issues must still be faced in this yet emerging scientific field. In conclusion, we tried to put together some major guidelines for mental health mobile app development in the context of psychological treatments.

## 1. Introduction

The use of mobile technology in health interventions has been named mHealth. This consists of all clinical practices that include the use of mobile devices for health purposes. The growth of smartphones and mobile applications (apps) has been widespread over the last decade and received a further boost during the COVID-19 pandemic.

Research over the last three years has shown the pandemic’s dramatic impact on mental health. Recent data have shown an increase in the emergence of loneliness, anxiety, depression, insomnia, harmful alcohol and drug use, and self-harm or suicidal behavior [1] because of the extreme health measures that the governments had to take to prevent the spread of COVID-19.

The lockdowns that many institutions chose to apply as a social distancing measure led to an increase in cases of domestic violence, since victims who had to share their space with domestic violence perpetrators had no escape from their abusers during quarantine [2].

The domain of emotional dysregulation appears, in this scenario, to be among the most impacted in mental health, since each of the aforementioned disorders brings increasing difficulty in the management of difficult emotions that lead to violent or harmful behaviors in the worst cases [2,3].

The same crisis has led health professionals and researchers to look at new ways to provide access to mental health services or improve traditional procedures while paying specific attention to the unmet needs of socially or economically disadvantaged people by taking advantage of technology to produce cost-efficient and scalable methodologies of intervention to face the limitations that come from emergency situations such as the one the world has faced over the last three years [4].

In this context, it would seem a reasonable strategy to focus on the use of mobile health (mHealth) apps, and one that promotes the development of new means to help mental health care systems to provide new effective possibilities for the care and cure of mental disorders [5].

Put simply, mHealth generally refers to the use of mobile apps for health or clinical purposes [6]. In app stores and the literature, we can find different typologies of mHealth apps according to their purpose. Apps have been designed for different goals and can generally be classified as those that (a) manage clients’ data and health services in large and small scenarios, from private practice to hospitals; (b) track and report clinical parameters and make diagnoses; (c) educate and provide information about health and specific medical conditions by translating medical terms for patients; (d) are for self-help use, especially in the psychological field; and (e) integrate with clinical treatment to train and improve skills that are important for good outcomes in a specific health domain [7]. This last category is the one on which we focus in this systematic review, which considers the studies that include mHealth apps as an adjunct to psychological interventions for emotional dysregulation.

One of the richest categories of mHealth apps deals with mental health, counting more than 10,000 apps available to users worldwide [8]. There is also growing evidence for the potential of mobile health apps in the management of mental health problems, which has aroused clinical and scientific interest in the field. Meta-analyses on mental health apps seem to have supported their efficacy at least for the three domains of anxiety, depression, and suicidal intentions [8]. Although recent research has highlighted these opportunities, many challenges remain open [9,10,11]. Some examples of ongoing issues in the current research include poor data governance and data sharing practices, clinical safety concerns over the management of adverse events and potentially harmful content, low levels of user engagement and the possibility of “digital placebo” effects, and workforce barriers to integration with clinical practice [7,9].

The extreme fragmentation of apps also induces some more difficulties in defining the best methodology for evaluating their efficacy. That is why some authors have been proposing new ways to frame mental health apps, such as through user-adjusted analysis, which faces the problem of dissemination by evaluating what specific aspect of app content is really engaging clients. In this wide scenario, the development of mHealth apps is a promising option that could increase the dissemination of evidence-based mental health care [12]. When considering methodological issues, we address both overall content quality and efficacy in improving therapeutic outcomes. In this paper, we focus more on the second methodological issue, considering the way that mHealth apps can affect, positively or negatively, the course of psychotherapy when added as an empowerment tool for psychotherapy practice.

Many mHealth apps incorporate key elements of evidence-based approaches, such as cognitive behavioral therapy (CBT) [13], acceptance and commitment therapy (ACT) [14], and dialectical behavior therapy (DBT) [15]. Each app makes use of these models in different ways, while clients take advantage of the features that are included in these apps in their own ways. In this promising yet challenging territory, mHealth apps can be used as standalone tools for self-help or as an adjunct to psychological treatments. This systematic review focuses on this latter case. Research examined for this review shows that the use of mHealth apps with human support is more effective and engaging than that of apps that are used on their own. We find in this paper that engagement has a core role in the effectiveness of this type of technology in the clinical setting, and it is one of the most severe vulnerabilities of smartphone technologies. In this work, we wanted to review the state-of-the-art studies that have included mobile apps as a means of augmenting psychological treatments that focus specifically on emotion dysregulation.

To accomplish this synthesis in the present paper, we have searched through the PsycInfo, PubMed, Web of Science, and Cochrane Library databases to access the scientific literature to be considered for a systematic review on this topic. At the core of this paper is the aim to consider the positive impact of mobile technology applied in traditional psychological treatments. This review follows a human-centered approach, with the relationship with the therapist having a central role. From this perspective, mobile apps are considered an empowerment tool in reducing sufferance and improve people’s health in the easiest way that the current technologies enable.

## 2. Materials and Methods

A comprehensive literature search was conducted of the bibliographic databases of PubMed, PsycInfo, Web of Science, and the Cochrane Library for relevant articles published from 1 January 2017 to 31 August 2022 (covering a period of five years of research). Terms indicative of mobile apps and mental health disorders were used to search these databases, with the search being limited to “humans”, English, and peer-reviewed journals. Full-text copies of all potentially relevant papers and papers whose abstract was insufficient to determine eligibility were obtained. The full-text articles were screened and discarded if they did not meet the inclusion criteria. References to earlier reviews and the included primary articles were also examined.

We included abstracts describing apps for mobile devices (mobile phones or tablets) that were used in a blended form within psychological treatment or psychotherapy and focused on emotional regulation. Symptoms and disorders considered in the selected literature included depression, anxiety, bipolar disorder, borderline personality disorder, post-traumatic stress disorders (PTSD), and suicidal behaviors. Studies examining the effects of mental health apps on recognizing the symptoms or disorders were also included. We chose to concentrate on adults and adolescents and placed no restriction on participant gender. Furthermore, we excluded other reviews and meta-analyses and included only feasibility, usability, and clinical trials.

In this systematic review, we focused very specifically on apps that had been developed and implemented in clinical settings with the support of human intervention. This choice was taken because research has shown that this kind of app is more effective in terms of engagement and the overall validity of the intervention. Moreover, the main focus of this review is to summarize the state of the art of this specific type of app. The apps we selected had been developed to enhance in different ways emotional regulation and to give an empowering tool to patients for developing skills to manage their symptoms.

We excluded abstracts if (a) the paper did not clearly describe the app; (b) the intervention was an Internet-based intervention, virtual reality exposure treatment, interactive voice response technology intervention, or a text messaging-only intervention without a mobile app component; (c) the study assessed interventions but not apps; (d) the paper provided solely a description of the mobile app or concept and no outcome data; (e) the intervention targeted other medical conditions other than mental health symptoms or disorders; (f) the intervention was not focused, among other elements, on emotional dysregulation or mood dysregulation; (g) the app was used without a psychological intervention; (h) the app was only used as an emotional momentary assessment (EMA) tool; and (i) the paper was not written in English.

One author (FD) selected the studies using the inclusion and exclusion criteria. Any issue during this process of selection (19 articles) was resolved by the other authors (LSS and MP).

## 3. Results

### 3.1. Selection and Inclusion of Studies

**Figure 1:**
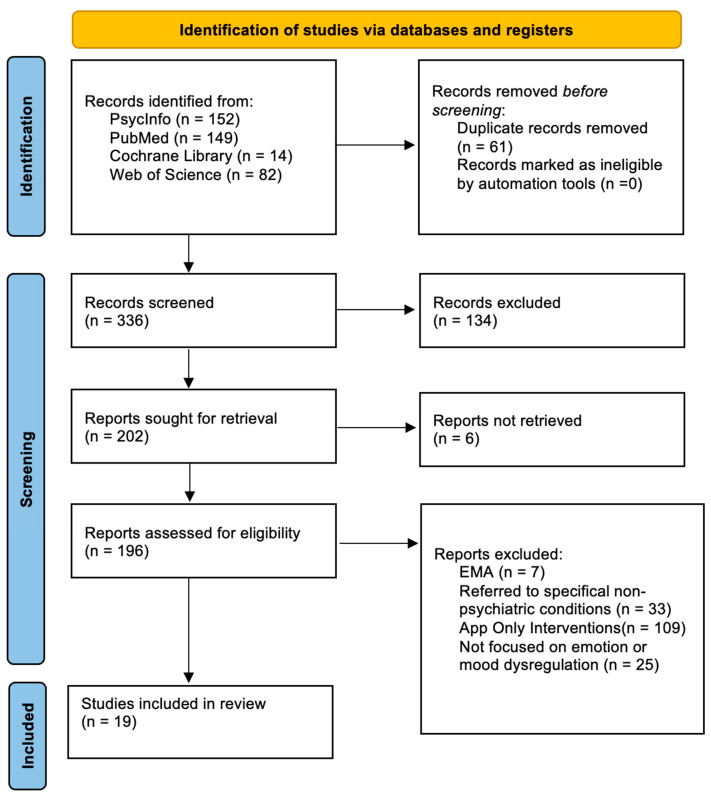
The PRISMA flow diagram summarizes the methodology in terms of identification, screening, and inclusion criteria used for this systematic review.

Of the 397 abstracts initially identified, 101 were excluded from this review based on their relevance to the topic after reviewing their abstracts, and another 61 were excluded because they were duplicates. Of the remaining 235 articles, 6 were not retrieved and 208 were excluded because they did not meet the criteria. Finally, a total of 19 full-text articles met the inclusion criteria for the current systematic review. Figure 1 presents a flow diagram detailing the review process and results at each stage.

### 3.2. Characteristics of the Included Studies

The 19 publications included in this review consisted of 3 feasibility studies [16,17,18], 6 randomized controlled trials [19,20,21,22,23,24], and 4 papers analyzing usability [25,26,27,28]. The other included studies were classified as four open trials [29,30,31,32], a pre- and post-intervention study [33], and a longitudinal qualitative study [34].

Publication dates ranged from 2017 to 2022. Table 1 reports the selected studies’ characteristics, including outcome(s), objective and design, sample enrollment method, and app name.

### 3.3. Validity of the Mental Health Apps

In this systematic review, we focused very specifically on apps that had been developed and implemented in clinical settings with the support of human intervention. The choice to do this was made because research has shown that apps such as these are more effective in terms of engagement and the overall validity of the intervention. Moreover, the main focus of this review was to summarize the state of the art for this specific type of app. The apps we selected had been developed to enhance in different ways emotional regulation and to provide an empowering tool to patients to develop skills to manage their symptoms. In the following paragraphs, we describe the findings and observations of the examined studies by dividing them into each of the diagnostic categories that were considered in the included papers.

### 3.4. Theoretical Framework

All apps included in the examined papers were based on theoretical frameworks that belong to or are derived from CBT [35]. The cognitive behavioral approach is a psychotherapy model focused on the collaboration between the patient and the therapist to discover the relationship between emotions, thoughts, and behavior [36]. Interventions focus primarily on reducing current suffering and then enabling work on any vulnerabilities in a profound way to minimize the risk of relapses. Shared goals are usually agreed at the beginning of the course, and the duration of the treatment is naturally based on achieving the agreed goals and on the patient’s feedback on their well-being [37].

A characteristic of this approach is the use of evidence-based procedures and techniques [38].

Over time, cognitive behavioral psychotherapy has integrated numerous contributions from other approaches, constantly improving its range of interventions and its effectiveness and proposing tools and work procedures both on the symptoms and on the deep dynamics of psychic functioning [39].

To date, this form of psychotherapy has been recognized for many disorders as an elective treatment with an efficacy over time that is greater than or equivalent to pharmacological treatment [37,40].

The two main derivations of CBT that authors have been shown to prefer for mobile app development are DBT and ACT. DBT was first developed for borderline patients and is mostly focused on integrating CBT principles with acceptance, mindfulness practices, and skills training to address emotional dysregulation and self-harming behaviors and to develop more cognitive and emotional flexibility [41,42,43]. ACT, too, is an upgraded form of CBT that focuses on mindfulness and acceptance practices to enhance psychological flexibility, and it focuses on personal values and their pursuit [44].

All of these approaches either integrate or are often paired with mindfulness practices. The term “mindfulness”, from a clinical point of view, refers to meditations and exercises that aim to improve the ability to be aware of the present moment through sensations, emotions, and thoughts to promote more aware and conscious behavior [45,46].

### 3.5. Mobile App Characteristics

All the mobile apps were described in the 19 included studies. Of these apps, six were for the management of emotional regulation and DBT skills for BPD patients [21,25,26,28,29,34], three were for the prevention of suicidal behaviors and non-suicidal self-injury (NSSI) [20,23,30], five were for the management of or psychoeducation for depressive and/or mood disorders [17,19,24,31,32], three were for the management of anxiety and depressive symptoms [16,18,27,33], and one was for PTSD anger management [22]. The paper by Weintraub and colleagues [32] also included psychotic spectrum patients and so it did not belong specifically to the emotion dysregulation domain of psychopathology. However, it was included because it mainly focused on intervention for patients with mood disorders.

In the following paragraphs, we provide complete descriptions of the apps’ features. These are divided by specific disorder or symptoms domain, since in many cases, the included apps that focused on a specific disorder shared common characteristics.

#### 3.5.1. Borderline Personality Disorder

Borderline personality disorder is among the most prevalent personality disorders in the general population, and it represents one of the most difficult disorders to treat. Moreover, it is often related to suicidal behavior and self-harm. Among the many different treatments that have been developed for this disorder, DBT is one of the most empirically supported for its effectiveness [42,43].

All the studies we examined in the sphere of BPD described DBT-based apps. Austin and colleagues [29] completed a feasibility study with 24 BPD patients in which they developed and tested a DBT-based app to be used together with psychotherapy. The app included some key features of DBT, such as psychoeducation, recording and monitoring of mood, descriptions of and guidelines for different behavioral strategies to deal with difficult emotions, and notes for when the therapist could not be present. The participants completed a semi-structured interview at the end of the study to assess their experience with the app from a qualitative point of view. The majority were comfortable with the app, and it was perceived to be a positive adjunct to therapy. The participants reported that it helped them to reinforce content acquired through therapy sessions and increased self-monitoring. The use of the app was also positively perceived in collaborations between patients and therapists, in contrast with common concerns about the impact of technology on therapeutic alliance. The drop-out rate was around 10%, which is less than the usual drop-out rate in DBT treatment with this kind of population. Some issues emerged concerning broad variability in app engagement, which is one of the most common issues for mHealth apps.

In another study by Derks and colleagues [25], the authors focused on emotional awareness through the use of a biofeedback app that aimed to enhance the ability of BPD patients to monitor their emotions from a physiological point of view. The app was developed for Android devices and included a smartwatch app that could measure heart-rate variability and electrodermal activity. The authors evaluated usability through a system usability scale, and the average score was between “OK” and “acceptable”. The app was provided to patients who were already under treatment with DBT. What emerged can be summarized as a request for more data visualization, more compatibility with the framework of therapies used, and the addition of notes for the diary. The study was conducted with two iterations, which included focus groups and modifications to the app following directives from the patients. A fundamental addition was the implementation of notifications, which patients reported to have increased their self-awareness.

Frias and colleagues [26] conducted another usability study with 25 participants assessing their satisfaction with and emotionally evaluating a mobile app for use together with psychotherapy that focused on third-wave CBT strategies to manage the strong emotions of BPD patients. The results showed the app to be perceived as useful at critical moments, and the overall satisfaction rate was quite high. The emotional evaluation of the app revealed a high global score, and it was found to be soothing, confidence inspiring, attractive, and enjoyable. These characteristics seemed to be important for pushing engagement in the use of the app, which was overall considered to be usable with borderline patients.

In the study by Laursen and colleagues [21], the authors focused on the use of a digital journal relative to a paper diary. This study involved 78 participants in treatment with DBT delivered by a therapist. Both groups of the RCT improved in all symptom domains, but the app group was shown to have better improvement in skills (measured by recording the number of times patients used DBT skills during the week), major improvement in quality of life, and a decrease in depression severity, borderline severity, and suicidal behavior. The study also showed a considerable economic difference between the two groups, since the cost for the app group was three times the average for the DBT-only intervention, introducing a negative aspect that should be taken into account when considering implementing mobile apps in psychotherapy services.

Prada and colleagues [28] focused on the development of a mobile app for the management of aversive tension in borderline patients. The app was specifically designed to address emotion dysregulation and impulsive behavior and was based on the principles of DBT to be integrated with psychotherapy. Sixteen participants showed self-harming behaviors and were already in treatment with DBT. The app that the authors developed included visual analog scales to monitor emotional tension and easy techniques and exercises to manage disruptive emotions. The focus of the EMOTEO app was on the generalization of skills in the natural environment of patients outside their therapy sessions. The study of Prada and colleagues [28] found that usability, satisfaction, and acceptability showed promising results among patients and seemed to confirm that those who used mobile apps as an adjunct to psychotherapy showed a higher degree of satisfaction with the treatment. The authors also noticed that the time of day when the participants used the apps matched with the time of day when most emotional crises occurred, and this was considered evidence that patients actually used the app in times of crisis, supporting the idea that one of the key features of these digital tools is to provide support 24/7 and in any situations when the therapist may not be available.

In the final study we examined in this category, Schiffler and colleagues [34] described a longitudinal qualitative study on the use of a DBT-based app for borderline patients. The duration of the study was 30 days, and participants were assessed through semi-structured interviews at the beginning and at the end of the testing period, during which they used the app. The participants consisted of 13 patients in treatment with DBT. The app did not allow patients to make entries, but it worked instead as a psychoeducational tool. The interviews mainly focused on suicidality and NSSI, and the qualitative results highlighted some important feedback from the patients, which was mainly that they felt safer by having all information related to skills, which is a core element of DBT, in their mobile devices, and this emerged as a supportive element for the time spent between psychotherapy sessions. Thus, the provision of a DBT-based app seemed to be highly acceptable by borderline patients, and there was a major preference for its efficacy in acute situations, suggesting that this aspect should be considered in UI design so that it is intuitive in emergency situations.

#### 3.5.2. Suicidal Behavior and NSSI

We found three papers describing mobile apps for suicide and NSSI prevention to be used together with psychotherapy. The first was the study by Beard and colleagues [30], which described the development of a mobile app to augment acute care for suicidal patients. Fourteen patients receiving CBT in a partial hospital were asked to participate in an open trial using a mobile CBT-based app that included exercises for performing cognitive reappraisal and bias modification. The study included feasibility, acceptability, and adherence measures concerning the app. Qualitative feedback was used to perform an iterative development of the app, and global acceptability ratings showed positive feedback in terms of satisfaction, perceived helpfulness, personal relevance, and user-friendliness. At the same time, the authors reported some negative feedback concerning exercise duration, desire for more variability, and flexibility.

Kennard and colleagues [20] developed a mobile app to reduce suicide attempts among adolescents. In this RCT, 66 adolescents were randomly assigned to the intervention program, the intervention program + app, or treatment as usual (TAU). The app was designed to give prompts to adolescents to rate their level of emotional distress and provided emotion regulation strategies to manage them, based largely on CBT and DBT principles. The intervention program was shown to be effective, the app was seen as acceptable and usable by the patients, but its presence did not seem to deliver any particular effect. Unfortunately, the study did not measure the aspects of the app and the intervention program that were the most active.

The third paper to focus specifically on suicide and NSSI was by O’Toole and colleagues [23] and described an RCT for an app-assisted intervention to prevent suicide among patients diagnosed with adjustment disorder who were in treatment. A total of 129 patients were randomly selected to receive TAU (N = 69) and TAU + app (N = 60). Overall, the latter group showed a smaller decrease in suicide attempts, showing it to be effective, but less than the control group (TAU). The authors stated that this effect may not be linked to a negative effect of the app, but to a smaller exposure to the treatment itself when replaced by use of the app. The mobile app used in this study contained psychoeducational materials, self-rating scales, reminders for daily entries about different parameters, and a “safety plan,” which described the actions to be taken in case of suicidal crisis. Other features included a collection of positive memories, a list of emergency contacts, and a selection of self-help exercises. An important aspect highlighted by the authors is the fact that there are no guidelines for the introduction of such technologies to patients, and this can lead to misinterpretation of the use of the app or to a scarce tendency to use it, and therefore engagement issues. One last consideration concerned timing, with the authors stating that more research was needed regarding the right time to use mobile apps, considering the effects of introducing technology at the beginning, end, and at different stages of the treatment.

#### 3.5.3. Anxiety/Depression

It seemed convenient to organize studies that focused on both anxiety and depression separately from those that dealt with depression and mood disorders, and it reflects the importance that depressive symptoms have as a target for app-enhanced interventions.

The first study we encountered that considered both depressive and anxiety symptoms was by Economides and colleagues [33]. In it, the authors assessed the impact of an 8-week program based on a mobile app as an adjunct to a psychological intervention to observe if the app’s presence could decrease the severity of anxiety and depression. The intervention was designed with a pre- and post- evaluation of feasibility, which seemed to have been successful, since the presence of the mobile app was positively accepted by the patients. Throughout the intervention, 102 participants were supported by a therapist while using the app, which was designed to integrate CBT principles to manage difficult emotional states. The participants reported a clinically significant reduction in depressive symptoms and also a significant improvement in anxiety symptoms. Clinical improvements were shown to be significant at the post-evaluation and also seemed to increase by 60% during 12 months of follow-up. Thus, the app showed promising results in terms of its efficacy from a clinical point of view.

The second study that focused on anxiety and depression was conducted by Levin and colleagues [18]. This paper focused on an ACT-based mobile app as an adjunct to acceptance and commitment therapy to improve psychological flexibility. Fourteen depressed and anxious adolescents used the app for two weeks in an open trial study with a pre- and post-evaluation. The mobile app used for this study was based on the ACT approach [41] and on ecological momentary interventions, which means that the app provided momentary assessments during the day, which were used to guide the participants to use the appropriate technique or skill for that specific moment. The app included 28 skill coaching sessions divided into 4 ACT categories, namely acceptance, defusion, mindful awareness, and values. The study showed that 85% of the participants completed an average of one app check-in per day, while 69% completed an average of two check-ins, which can be considered as good results in terms of engagement. On the feasibility side, the authors highlighted that the participants criticized the repetitiveness of the app’s content, wishing for more of it, while skills material contained too much written text, supporting the importance of clear and simple UIs. In terms of efficacy, the participants showed improvements both in depressive and anxious symptoms and in the psychological flexibility area. The authors concluded with some suggestions, the first being to implement a tailored approach to the app, giving as much freedom of personalization as possible in terms of the way the app should work for specific patients. The second was to improve the algorithm that the app relied on for providing suggestions about skills and techniques linked to the momentary assessment. Third, the authors reported it to be a challenge to find collaborative therapists who were willing to participate with their patients in this study, revealing difficulties and challenges related to the likelihood of therapists and psychologists to accept and use such technological tools.

The third study we considered was by Broglia and colleagues [16] and described the feasibility study of an app-supported counselling intervention for 38 university students. The feasibility trial used a two-arm, parallel non-randomized design with a group following TAU + app and the other with TAU. All participants were diagnosed with anxiety or depressive symptoms. Both groups reported improvements in terms of symptomatology after 6 counselling sessions, but at 6 months follow-up, the participants in the TAU + app group were shown to have continued decreasing symptoms. The app was considered to have been positively received by participants, which demonstrated its feasibility as an adjunct to counselling. The app included several features based on CBT and allowed participants to monitor daily behaviors, cognitive restructuration, guided relaxation, anonymous online groups, and goals tracking.

The final study to focus specifically on anxiety and depression was reported by Newton and colleagues [27]. The authors described the implementation of a CBT-based app for between-sessions training as an adjunct tool for psychotherapy treatment. Ten adolescents who were in treatment with three therapists were provided a mobile app developed to include homework that was coherent with the treatment they had been following and with the ability to be monitored and to provide rewards from their therapists. The study was designed into three phases. The first phase included the app design and development; the second phase involved testing the app outside treatment; and in the third phase, the app was tested with patients in treatment for 13 months and collected data as case studies. The authors described some emerging guidelines for developers, including an autonomy-based approach, allowing patients to tailor their experience based on the treatment they had already received, the app’s ease of use, and the link between this aspect and the engagement in the use of the app outside sessions. Patients and therapists showed appreciation for the use of the app, and it seemed a promising tool to help patients in between sessions.

#### 3.5.4. Depression/Mood Disorders

Among the most frequent diagnostic targets we encountered in this systematic review were depression and mood disorders. This should be no surprise since depressive symptoms are among the most widely presented in the general population and represent the lower side of the emotion dysregulation spectrum.

The first paper we examined was by Dahne and colleagues [17], and it described the feasibility study for a mobile app developed to train patients in behavioral activation while they followed ongoing psychotherapy. The mobile app included a diary for mood and behavior monitoring; inventory for life areas, values, and activities; a schedule for values-based activities; and an “assist” section to provide useful strategies for accomplishing tasks that were part of the behavioral activation program based on specific activities the patient was committed to perform in order to improve his/her mood. The app also included a rating scale for daily mood monitoring. The psychotherapists were provided PC software to monitor the patients’ data that included mood graphs and access to session materials.

The research included 10 psychotherapists and 11 patients and was developed as an open trial study. The results showed that patients improved over time on baseline depression and symptoms. The overall feedback from the therapists and patients was positive with regard to the acceptability and feasibility of the app. The authors highlighted that the large amount of information provided on the patient’s activity could present an opportunity to intervene as fast as possible on the issues and difficulties of patients.

Dahne and colleagues performed a second study [19], this time of a different mobile app. In this study, the authors used a pilot randomized trial methodology to observe the acceptability of a CBT-based app to support depressed patients during treatment. App usage retention was quite high during the trial duration of 8 weeks, with 70% active users, which slightly reduced to 50% after 1 month from the enrollment. This study highlighted once more the engagement issues that these tools encounter when integrated into clinical practice.

In the study by Miklowitz and colleagues [31], the authors investigated the clinical effects of a mobile-enhanced family-focused therapy among adolescents at risk for mood disorders. The mobile app included 12 lessons that were part of the program and included family communication, problem-solving, and mood management. The program had a duration of 18 weeks and involved 22 adolescents and their parents. The results showed acceptable engagement scores, the overall acceptability of the app was good, and it was perceived to be a positive tool.

Another selected paper described an RCT with 124 adolescents and focused on an 8-week CBT-based program conducted by therapists for depressed patients and supported by a mobile app. The authors [24] designed the study with one group receiving TAU and the other receiving TAU + app. The intervention and control groups resulted in not significant differences, with both experiencing efficient symptoms reduction. The program included elements from CBT, mindfulness-based programs, and behavioral activation. Unfortunately, the paper did not describe the structure and functions of the app.

The final paper belonging to this category focused on mood and psychotic disorder [32]. We thought to include it because it mostly focused on the emotional regulation features of the app and was compatible with all the other criteria we used for this systematic review.

This study focused on a CBT protocol for severe mood and psychotic disorder (Unified Protocol for Adolescents) and was designed to be provided to 24 patients in a 9-week program. The intervention was augmented with the use of a mobile app that was designed through a user-centered approach to generalize and extend the sessions’ content by reviewing it and practicing treatment skills.

The authors used focus groups to analyze participants’ feedback and used this information to improve the implementation of the app. The outcomes of the study included engagement, which was measured through accessing the app, and were shown to be acceptable overall. The app was indeed considered satisfactory by patients, and it was feasible for use together with human intervention. One of the key outcomes that the authors reported was that a greater skill practice via the app was associated with a greater improvement in mood and cognitive functioning.

#### 3.5.5. Post-Traumatic Stress Disorder

We found only one study related to PTSD based on the inclusion criteria of this review, which was by Mackintosh and colleagues [22], and it focused on anger management in 58 veterans.

The authors described an RCT with a control group receiving a 12-session program for anger management based on CBT, while the intervention group had the same intervention adjunct with a mobile app. The app was integrated with companion desktop software for therapists and a wearable device to monitor heart rate. There were found to be no differences in outcome between the groups, while the participants in both treatment groups demonstrated statistically significant and clinically meaningful reductions in anger severity and significant post-treatment reduction in PTSD.

## 4. Discussion

Having assessed the different studies we identified in this systematic review, we can try to gather some considerations about the current state of the art in this field.

Most of the research was conceptualized as RCT or feasibility studies. It is clear that at this stage, studies considering mobile apps as an adjunct to psychological treatment are still in their infancy. Considering the current literature, what we can assume is that the authors were most interested, until the time of this review, in understanding if this technology was useful for patients.

We deliberately excluded studies that focused only on EMA because we were interested in solutions that could directly affect the treatment, rather than just providing information to clinicians.

Patients seemed to positively accept the use of mobile apps to support their treatment, and in all of the studies, we found a good response in this sense. In the RCTs, we found that the intervention group often saw the most improvement over time, and the presence of apps seemed to expand the duration of improvement over time in the follow-up.

In other cases, the intervention groups did not show a significant difference with the control groups, despite demonstrating their efficacy. This is an interesting aspect to consider, since this may have been due to the fact that the TAU was very effective per se, so the adjunct of a mobile app could not be appreciated. Beyond this consideration, it is evident that there still is no evidence of which therapeutic factors are most affected by the use of mobile apps, and this lack of evidence makes it difficult to understand what exactly the apps contribute to the therapeutic process. In our opinion, this is one of the most important features of this technology that is yet to be discovered, since it can dramatically impact the design and implementation of mHealth apps.

Considering the literature of this systematic review, we can hypothesize that some of the possible therapeutic factors impacted by the use of mobile apps include agency, skills generalization, working alliance, and emotion management.

Mobile apps may improve agency because they give patients the ability to manage themselves in different ecological situations, where having “therapy in their pockets” could improve their sense of efficacy in performing the right actions, taking advantage of reminders, cognitive restructuration, suggestions, and other active material. Skills generalization may be increased, since patients have constant access to all the information they need to serve them in their daily life, and the more that skills are used, the more they are generalized to different situations and become positive habits. Considering the literature [47,48], even the working alliance could benefit from the adjunct of mobile apps, since their introduction is an act of trust toward the patient, and at the same time, they act as a bridge between sessions, increasing the mental representation of the clinician and the therapy outside sessions, and this could positively influence the therapeutic bond, reminding patients that the psychologist is present with them. Last but not least, mobile apps can provide the tools to manage difficult emotions right when they happen and are less directly addressable by the therapist. They are linked directly to what the patient and the clinician do in sessions, helping patients to feel less alone and more confident in managing their emotions.

The examined literature seemed to suggest that the integration of mobile apps with human support can be greatly profitable in terms of engagement. Excluding some isolated cases, engagement was well maintained in most of the examined papers, and this aspect may confirm the evidence that mobile apps show engagement issues mainly when they are not used as an adjunct to treatment. This aspect is coherent with any compliance study that supports the idea that a working alliance is a fundamental factor to affect the use of medication or any behavioral change [49,50]. We could imagine that mHealth apps are like medications; patients use them and gain advantages from them when there is therapeutic compliance that cannot be regardless of human support.

The potential of mHealth apps passes through engagement, and engagement in treatments cannot happen without a working alliance with the clinician. At the same time, alliance is the most powerful factor in good psychological treatment [51,52], so mHealth apps must have the core goal of amplifying the working alliance and strengthening it. That is why it would be desirable to see studies on the impact of mHealth apps on working alliances in psychotherapy and psychological treatments.

## 5. Conclusions

The current review aimed to summarize the state of the art on the adjunct of mobile apps to psychotherapy to facilitate emotion regulation. To the best of our knowledge, this is one of the first systematic reviews that focuses on emotional dysregulation and interventions through mobile apps to augment psychotherapy. Most of the studies we identified focused on standalone mobile apps, as they are the most common to find on the Apple App Store and the Google Play Store.

We began this review, which is focused on the blend of human interaction with technological support, on the basis of research evidence that shows that the adjunct of mobile mental health apps is more effective than mobile apps alone. There is yet more research to be conducted to better support the preliminary evidence that mHealth apps can improve the effectiveness of psychotherapy. What emerges from this review is that mHealth apps seem to be more studied as adjuncts to psychotherapy for treating more severe disorders, such as borderline personality disorder or depressed patients with high risk of suicidality, than less disabling conditions. We believe that this systematic review can be a starting point for more research on this very young field. We imagined this review from a clinical point of view, which means that we wanted to understand if mobile apps have been shown to be feasible and acceptable, while not proven to be effective, as an adjunct tool for psychological interventions. We were not interested in standalone mobile apps without therapy exactly for this reason.

The current systematic review suggests that mobile apps for mental health can provide clinicians potentially feasible and acceptable tools that can be effective in monitoring and improving the symptoms of certain mental disorders, helping patients to generalize skills, improving learning about symptoms, and thus delivering a more aware approach by patients. mHealth apps have been shown to be useful, from a preliminary qualitative overview of the examined literature, in improving patients’ agency and sense of empowerment, giving them concrete tools to manage emotion dysregulation outside the therapist’s room.

Given the widespread usage of smartphones and tablet devices among the general population, especially among younger people, mHealth has enormous potential to be an effective tool in mental health care delivery, and we think that it would not be smart to miss the opportunity to use this pervasive technology in mental health practice. This desire for a more accepted synergy between clinical psychology and this technological field could be beneficial both for patients and clinicians by providing more accessible solutions to improve psychological treatments and at the same time by reaffirming the central role of the psychologist and psychotherapist in delivering this kind of intervention.

As we mentioned in the introduction of this paper, the COVID-19 pandemic has exposed the general population to a potentially collective trauma and an outbreak of mental health issues. But from such a difficult and dramatic situation, we can try to take the opportunity to rethink the way that mental health is considered and develop new therapeutic interventions by taking advantage of current and future technology to reach the general population more easily, more quickly, and more efficiently.

In the future, we may imagine mobile apps developed with rigorous standards and an evidence-based approach applied directly to apps. This consideration comes from having observed that even if many apps are based on scientifically solid frameworks, such as DBT, CBT, ACT, and mindfulness-based interventions, this does not automatically mean that the apps are even valid. It is a wish for the future that mobile apps will be more than simply digital twins of self-help books and instead be tools that can really leverage technology to be more interactive and pervasive in a good and healthy way.

Our aim and vision are not to replace the psychologist with a mobile app, which would be a loss in terms of clinical efficacy, since we know the importance of interpersonal factors such as working alliance, therapeutic relation, corrective emotional experience [48,49], and many more aspects that research has shown to be linked to successful psychological interventions for which a human presence is necessary.

Our goal is to seize the opportunity to introduce mature technology into psychological treatments, upgrading the “paper and pencil” approach with feasible and useful tools that must be developed, designed, and tailored by psychologists for psychologists and patients. We hope that this review will be a starting point to develop clinical solutions and research in this vast, yet still scarcely explored, scientific field.

## Figures and Tables

**Table 1:** Characteristics of publications included in the review (N = 19).

Study	Target	Objective and Design	Sample	App Name
Broglia et al. (2019) [16]	Anxiety, Depression	Feasibility study: use of a mobile app as an adjunction to a counselling service for university students. Methods included the comparison of TAU and TAU + App. Feasibility factors were evaluated through clinical evaluations in sessions and in post-intervention follow-ups.	N = 38, students aged over 18 from a university counselling service	Pacifica
Dahne et al. (2018) [17]	Depression	Feasibility study: development and initial evaluation of a behavioral activation app in conjunction with psychotherapy. Feasibility factors were evaluated monitoring symptoms outcomes and qualitative therapists’ and patients’ feedbacks.	N = 10 therapists recruited from a clinical psychology doctoral program, and N = 11 patients recruited via e-mail	Behavioral Apptivation
Levin et al. (2017) [18]	Depression, Anxiety	Feasibility study: adjunction of a mobile app in acceptance and commitment therapy to improve symptoms and study acceptability. Pre- and post- evaluations were used to assess participants’ satisfaction within the use of the app.	N = 14 patients currently receiving ACT	ACT Daily
Dahne et al. (2019) [19]	Depression	Randomized controlled trial: comparison between behavioral activation program, BA + App and TAU.	N = 52 patients recruited from primary care practices	MoodKit
Kennard et al. (2018) [20]	Suicidal behavior	Randomized controlled trial: use of a mobile app to support an intervention for the reduction of suicidal attempts following hospital discharge. One group was administered with TAU, while the other with TAU + App. A blind evaluator assessed the suicide risk at different follow-up times for each group.	N = 66 adolescents, hospitalized for suicidal ideation (N = 26) and recent suicide attempts (N = 40), aged 12–18, recruited as psychiatric in-patients	BRITE
Laursen et al.(2021) [21]	BPD	Randomized controlled trial: economic evaluation of the use of a mobile diary vs. paper-based diary card with patients treated with DBT.	N = 78 patients currently receiving DBT treatment	mDiary app (Monsenso System)
Mackintosh et al. (2017) [22]	PTSD	Randomized controlled trial: comparison between anger management treatment with AMT augmented with a mobile app or TAU.	N = 58 war veterans with PTSD symptoms recruited through the department of Veteran Affairs	RELAX App
O’Toole et al. (2019) [23]	Suicidal behaviors	Randomized controlled trial: testing the use of app-assisted treatment for suicide prevention. The group with TAU and the other group with TAU + App were assessed considering symptoms during treatment and at a 4-month follow-up.	N = 129 participants, N = 69 with treatment as usual, and N = 60 with TAU + APP recruited between patients in psychotherapy treatment	LifeApp’tite
Raevuori et al. (2021) [24]	Depression	Randomized controlled trial: testing of efficacy of a therapist-guided intervention involving a mobile app with depressed patients. The group with TAU and the other group with TAU + App were assessed considering symptoms during treatment and at different follow-up times.	N = 124 women, mean age = 25, recruited from general practitioners from 11 cities in Finland	Not available
Derks et al. (2019) [25]	BPD	Usability study: development of an ambulatory biofeedback app for borderline patients to improve emotional awareness. Three cycles of testing were performed for patients, therapists, and UCD experts. Data were collected through questionnaires, interviews, and task scenarios.	N = 5 patients in treatment with DBT, aged 18–49	Sense-IT
Frias et al. (2021) [26]	BPD	Usability study: implementation of mobile app for emotional crises in borderline patients. Usability, satisfaction, and clinical symptoms were measured using questionnaires to evaluate the impact on participants.	N = 25 outpatients diagnosed with BPD	B-RIGHT
Newton et al. (2020) [27]	Anxiety, Depression	Usability study: development, design, and test of a smartphone-based intervention for adolescents with anxiety to use within CBT sessions. The first phase was dedicated to the development of the app. In the second phase, the feedback of patients was collected through interviews and questionnaires to improve the app. In the third phase, satisfaction and usability were assessed in patients and therapists.	N = 10 adolescents and 3 therapists	MindClimb
Prada et al. (2017) [28]	BPD	Usability study: development and implementation of a mobile app for emotional regulation in BPD patients in treatment with DBT. The efficiency of the app was evaluated for monitoring and reduction of aversive tension through questionnaires and interviews.	N = 16 BPD patients, all women recruited from a specialized treatment center for BPD	EMOTEO
Austin et al. (2020) [29]	BPD ^1^	Open clinical trial: description of participants’ experiences using a mobile app to enhance dialectical behavior therapy for borderline patients. Questionnaires and interviews have been administered to describe patients’ experiences with the app.	N = 24, recruited from psychiatric outpatient clinics	Not available
Beard et al. (2021) [30]	Suicidal behaviors	Open clinical trial: researchers collected data about acceptability and adherence during acute care for patients who completed a partial hospitalization to evaluate the usefulness of the app through iterative development.	N = 14, aged 19–64, people attending partial hospital	HabitWorks
Miklolwitz et al. (2021) [31]	Mood disorder	Open trial: integration of psychosocial intervention with mobile app in family-focused therapy for mood disorders. Adolescents and parents expressed daily ratings on patients’ mood and parents’ criticism. Independent evaluators then interviewed adolescents at follow-up times to track symptoms.	N = 22 adolescents, mean age 15.4, 45.5% female and their 34 parents	Not available
Weintraub et al. (2022) [32]	Mood disorders, psychotic spectrum disorders	Open trial: implementation and integration of a psychoeducational mobile app to use between sessions in group treatment for mood and psychotic disorders.	N = 24 adolescents, mean age 15.2, and their parents	Not available
Economides et al. (2019) [33]	Anxiety, Depression	Pre- and post-intervention study: impact on symptoms reduction with a smartphone-delivered and therapist-supported 8-week intervention.	N = 102 adults recruited through Facebook advertisements	Ascend
Schiffler et al. (2022) [34]	BPD	Longitudinal qualitative study: implementation of a mobile app and its impact on suicidal behavior and NSSI. Semi-structured interviews were administered for 30 days to assess the attitude towards the app.	N = 13, aged 18–23, diagnosed with BPD and former patients from a transitional psychiatric hospital	DBT app

^1^ Abbreviations: PTSD, post-traumatic stress disorder; BPD, borderline personality disorder; TAU, treatment as usual; DBT, dialectical behavior therapy; ACT, acceptance and commitment therapy; CBT, cognitive behavioral therapy; NSSI, non-suicidal self-injury.

## Data Availability

No new data were created or analyzed in this study. Data sharing is not applicable to this article.
